# Predictors of macular pigment and contrast threshold in Spanish healthy normolipemic subjects (45–65 years) with habitual food intake

**DOI:** 10.1371/journal.pone.0251324

**Published:** 2021-05-27

**Authors:** Begoña Olmedilla-Alonso, Elena Rodríguez-Rodríguez, Beatriz Beltrán-de-Miguel, Rocío Estévez-Santiago, Milagros Sánchez-Prieto

**Affiliations:** 1 Department of Metabolism and Nutrition, Institute of Food Science, Technology and Nutrition (ICTAN-CSIC), Madrid, Spain; 2 Department of Chemistry in Pharmaceutical Sciences, Faculty of Pharmacy, Complutense University of Madrid, Madrid, Spain; 3 Department of Nutrition and Food Science, Faculty of Pharmacy, Complutense University of Madrid, Madrid, Spain; 4 Faculty of Experimental Sciences, Francisco de Vitoria University, Pozuelo de Alarcón, Madrid, Spain; Université Clermont Auvergne - Faculté de Biologie, FRANCE

## Abstract

**Introduction:**

The dietary carotenoids lutein (L) and zeaxanthin (Z) are transported in the bloodstream by lipoproteins, sequestered by adipose tissue, and eventually captured in the retina where they constitute macular pigment. There are no L&Z dietary intake recommendations nor desired blood/tissue concentrations for the Spanish general population. Our aim was to assess the correlation of L&Z habitual dietary intake (excluding food supplements), resulting serum concentrations and lipid profile with macular pigment optical density (MPOD) as well as the contrast sensitivity (CT), as visual outcome in normolipemic subjects (n = 101) aged 45–65.

**Methods:**

MPOD was measured by heterochromatic flicker photometry, serum L&Z by HPLC, the dietary intake by a 3-day food records and CT using the CGT-1000-Contrast-Glaretester at six stimulus sizes, with and without glare.

**Results:**

Lutein and zeaxanthin concentrations (median) in serum: 0.361 and 0.078 μmol/L, in dietary intake: 1.1 mg L+Z/day. MPOD: 0.34du. L+Z intake correlates with their serum concentrations (rho = 0.333, *p* = 0.001), which in turn correlates with MPOD (rho = 0.229, *p* = 0.000) and with fruit and vegetable consumption (rho = 0.202, *p* = 0.001), but not with lutein+zeaxanthin dietary intake. MPOD correlated with CT, with and without glare (rho ranges: -0.135, 0.160 and -0.121, –0.205, respectively). MPOD predictors: serum L+Z, L+Z/HDL-cholesterol (β-coeficient: -0.91±0.2, _95%_CI: -1.3,-0.5) and HDL-cholesterol (R^2^ = 15.9%). CT predictors: MPOD, mainly at medium and smaller visual angles (corresponding to spatial frequencies for which sensitivity declines with age) and gender (β-coefficients ranges: -0.95,-0.39 and -0.13,-0.39, respectively).

**Conclusion:**

A higher MPOD is associated with a lower ratio of L+Z/HDL-cholesterol and with a lower CT (higher contrast sensitivity). The HDL-cholesterol would also act indirectly on the CT improving the visual function.

## Introduction

Macular pigment (MP), in the central region of the retina, comprises lutein and zeaxanthin, major carotenoids in the human diet, and meso-zeaxanthin, which is rarely found in diet and is believed to be formed at the macula [1]. Of the many carotenoids present in the diet and transported in the bloodstream to tissues by lipoproteins, only these are captured by the retina where they act as blue light filters, antioxidant, anti-inflammatory agents [2], and enhance gap junctional communication [3], which, in the retina, is important for light processing [4]. An adequate lutein and zeaxanthin status (serum concentrations and macular pigment optical density [MPOD], as short and long-term surrogate markers, respectively) is associated with a lower risk of several age-related diseases, particularly age-related eye diseases [5–7]. The relationship between MPOD and visual function has been mainly studied in subjects with ocular disease and/or those using lutein supplements [8, 9]. Although there are few studies in older subjects without ocular disease and none using lutein supplements, a relationship between macular pigment and several measures of visual function has also been described [10–12].

Lutein and zeaxanthin are among the food components that have proven to be effective in lowering the risk and /or progression of age-related macular degeneration (AMD), a major cause of blindness in the elderly population [13]. There has recently been an increase in research looking into the potential of lutein in slowing down age related retinal degeneration and damaged cognitive functions [2, 6]. Up to 10 mg lutein/day has been recommended for people at risk or in the intermediates stages of age-related macular disease [14], equivalent to approx. 150–240 g spinach (boiled or raw, respectively)/day. However, there are no lutein and zeaxanthin dietary intake recomendations for general population, nor are there desired blood or tissue concentrations despite ample information rearding their status, dietary intake and biochemical markers. This is partly due to the great deal of variability in carotenoid bioavailability which depends on several host and food related factors [15, 16], including n-3 fatty acids, docosahexaenoic (DHA) and eicosapentanoic (EPA) content [17, 18]. However, reference recommendations have recently been claimed for lutein intake as regards optimal ocular health and lowering the risk of chronic eye disease as this molecule meets a series of global historical criteria established for non-essential bioactive components regarding diet and health [19].

The macula is an important target tissue to consider when developing dietary recommendations for carotenoids, MPOD is the most accessible marker for xanthophyll concentration in the macula [16]. Moreover, evidence has been found supporting the effect of dietary supplements containing xanthophyll carotenoids in improving visual performance in patients and controls [12], DHA facilitates accumulation of lutein and zeaxanthin in the macula [7]. However, few studies with data on lutein and zeaxanthin concentration in blood and in the dietary intake assessed simultaneously in subjects with habitual food intake [16] and, few studies have focused on the visual outcomes of lutein and zeaxanthin habitual dietary intake and status (serum concentrations and MPOD) with visual outcomes (eg. contrast sensitivity, contrast threshold [CT], glare sensitivity, photostress recovery, visual acuity) in adults with healthy eyes [4, 12]. Previously, we described MPOD and CT age-specific correlations in apparently healthy subjects over 45 y but not in younger (20–35 y) subjects. The MPOD correlated with lutein plus zeaxanthin expressed in relation to serum lipid concentrations [20] and the CT correlated MPOD and serum lutein concentration [21]. In this study we aimed to deepen our understanding of previously observed age-specific correlations between MPOD and CT with lutein and zeaxanthin dietary and status markers and lipid profile in a larger sample of subjects within an age range 45–65 y. We evaluated whether such parameters could improve visual outcomes of the Spanish subjects.

## Materials and methods

### Design

Cross-sectional study analyzing lutein and zeaxanthn dietary intake, status of lutein and zeaxanthin by serum concentrations and MPOD, as well as serum lipid profiles–assesing their predictive value on the visual function of middle aged subjects.

### Participants

101 volunteers (77 women and 24 men) age 45 to 65 y (mean ± SD: 54.4 ± 0.6 y) enrolled in a cross-sectional study. Participants were selected among those interested after learning of the study through advertisements at different universities, research centers and notice boards. The inclusion of an equal number of women and men was not posible due to the lack of interest on the part of men. Nine out of the 101 recruited subjects were excluded because of repleted serum lutein concentration above 0.63 μmol/L, concentration considered as attainable by dietary means in normolipemic subjects [5, 20] as, higher serum lutein concentration has been described when lutein supplements are consumed [5].

The inclusion criteria were normal cholesterolemia (upper limit *ca*. 6.22 mmol/L), body mass index (BMI) ≥20 and ≤30 kg/m^2^, mixed diet (no avoidance of any food groups). Exclusion criteria (self reported): consumption of dietary supplements, myopia surgery within the previous year, cataracts, macular degeneration, use of drugs or phytosterol-enriched beverages/foods to control cholesterolaemia, consumption of n-3 fatty acid-enriched food products and chronic diseases that can affect carotenoid or lipid metabolism (i.e. diabetes, cardiovascular difsease). Of the one hundred and twenty one individuals who showed their interest in participating in the study, five were excluded because of his/her BMI, three because they were taking food supplements (omega-3, lutein+zeaxanthin), three because of their age, two because of statin consumption, two because of ocular chronic diseases, three without giving any explanation, one candidate followed a vegan diet and one because of gastric bypass.

This study was approved by the Ethical Committee of Research with Drugs of the Hospital Universitario Puerta de Hierro Majadahonda of Madrid, Spain (acta n° 03.17, dated 13 February 2017) and by the Bioethic Subcommittee- Ethics Commitee (CSIC) (dated 21 February 2017). Written informed consent was obtained from all subjects.

### Procedures

The volunteers included in this cross-sectional study underwent blood sampling, assessment MPOD and of the visual function, measured by the contrast senstivity, and 3-day food records. The subjects were enrolled over the course of an entire year (spring and summer: n = 49 and during the fall and winter: n = 62). Blood samples were collected after overnight fast (at least 9 hours).

#### Lutein, zeaxanthin and lipid analysis in blood

Lutein and zeaxanthin levels were determined by high performance liquid chromatography (HPLC) using a system consisting of a model 600 pump, a Rheodyne injector and a 2998 photodiode array (PDA) detector (Waters, Milford, MA, USA). The system included a C30 YMC column (5 μm, 250×4.6 mm i.d.) (Waters, Wilmington, MA) with a guard column (Aquapore ODS type RP-18). The mobile phase, in a linear gradient, was methanol (MeOH) with 0.1% triethylamine (TEA) / metyl tert-butyl ether (MTBE) from 95:5 to 70:30 in 25 min, to 35:65 in 25 min, to 95:5 in 10 min and maintaining this proportion for 8 min. The flow rate was 1 mL min^−1^, and detection was performed at a wavelenght of 450 nm. All chromatograms were processed using Empower 2 software (Waters, Milford, MA, USA). Identification was carried out by comparing the retention times with those of authentic standards and on-line UV-VIS spectra.

Carotenoid extraction was performed on serum samples using a slight modification of a previously published method [22]. Briefly, 600 μl of serum was added to 600 μl of ethanol, vortexed and extracted twice with 1200 μl of hexane: dichloromethane (5:1) stabilized with 0.1 g/L butylated hydroxyltoluene (BHT). Organic phases were pooled, evaporated to dryness under nitrogen atmosphere and reconstituted with 200 μl of a solution of MeOH: MTBE (1:1) and injected (50 μl) onto the HPLC system.

MeOH and MTBE from Honeywell Research Chemicals, triethylamine and butylated hydroxytoluene from Sigma-Aldrich, hexane and dichloromethane were supplied by Lab-Scan and Panreac (Barcelona, Spain), respectively. Lutein (xanthophyll from marigold) and zeaxanthin were obtained from Sigma-Aldrich.

Standard solutions were prepared from 1 mg of lutein and of zeaxanthin dissolved in 25 mL tetrahydrofuran, with 0.01% BHT in each case. The E _1cm_^1%^ values and wavelengths used were: lutein, 2550 at 445 nm; zeaxanthin, 2540 at 450 nm. Working solutions were obtained from different volumes of the standard solutions dissolved in MeOH: MTBE (1:1 v/v). The concentrations of the carotenoids in the curve were: 0.27–1.36 μg mL^−1^ for lutein (R^2^ = 0.999) and 0.03–0.15 μg mL^−1^ for zeaxanthin (R^2^ = 0.999).

Blood biochemical variables were analysis using the ADVIA Chemistry XPT System (Siemens Healthineers Spain). Blood total cholesterol were analyzed by enzymatic assay and and high-density lipoprotein (HDL) cholesterol by catalasa assay kit. Serum triglycerides (TG) were determined colorimetrically using the Fossati reaction with a Trinder type reaction final (GPO Trinder). Glucose was analyzed by the hexokinase method. The low-density lipoprotein (LDL)-cholesterol level was calculated with the Friedewald equation [23].

#### Dietary intake assessment

Recent dietary intake was evaluated using 3-day food records involving 24 h recalls, one of which coincided with a weekend or holiday, carried out within a period of 7 to 10 days. The participants underwent a face-to-face encounter with a specialized interviewer for the first recall and subsequently, performed the other two recalls by telephone. The amounts consumed were estimated in units (fruits), portions or household servings [24]. On the basis of this information, we calculated food intake in grams/day, which served as the basis for the determination of the daily lutein and zeaxanthin intake using a database [25] included in a software application for the calculation of dietary intake of individual carotenoids [26]. This carotenoid database contains data of the major dietary carotenids which have been studied in the relationship between diet and health (lutein, zeaxanthin, β-cryptoxanthin, α-carotene, β-carotene) in 124 foods, and all of them were generated by high-performance liquid chromatography. The food groups included in the software are: fruit, vegetables, oils and fats, eggs and egg products, milk and dairy products, snacks, nonalcoholic beverages and sauces and seasonings. To evaluate the energy intake, we employed a food composition table included in the software DIAL^®^ [24].

#### Macular Pigment Optical Density (MPOD) assessment

Macular pigment optical density was assessed using an MPS 9000 desktop device (Macular Pigment Screener, Elektron PLC, Cambridge, UK) that applies the principles of heterochromatic flicker photometry. The technique and reliability of this device are described in detail by van der Veen et al. [27]. The test consists of two stages for central and peripheral viewing, and the subjects were required to press a response button as soon as they detect flicker. The subjects started by fixating the central stimulus, a 1-degree central target (flicker rate is initially set to 60 Hz and then gradually reduced at a rate of 6 Hz s−1). The process was repeated for a series of green-blue luminance ratios. The observer then fixated a red 2°-diameter target placed 8° eccentrically and a second set of data were recorded for peripheral viewing.

#### Visual contrast sensitivity and contrast threshold

The contrast sensitivity is the inverse of the contrast threshold (CT). CT was measured with the CGT-1000 Contrast Glaretester (Takagi, Japan), which determined the CT by means of an automated strategy, set for 6 sizes of annular stimuli with diameters ranging from 6.3° to 0.7° of visual angle, with and without glare light conditions. There were 12 levels of CT, ranging from 0.01 to 0.45. The lower the CT, the higher the contrast sensitivity level at which a subject was able to detect each spatial frequency. Each subject was tested monocularly for CT, once with each eye and with spectacle correction when necessary.

The luminance of the background on which the stimulus was presented was 10 cd/m2. The specifications selected for the presentation of the stimulus were: presentation duration, 0.2 seconds; presentation interval, 2 seconds; test luminance with glare of 40,000 cd/m^2^; test distance, 350 mm. The device had 8 glare sources arranged around the stimulus that were activated automatically to assess the CT with a simultaneous glare. The test results were automatically printed out on a single graph that showed the sensitivity functions.

### Statistical analysis

Data are expressed as means and standard deviations, medians and 95% confidence intervals (CI). Lutein and zeaxanthin in serum and diet and TG did not follow a normal distribution (assessed by Kolmogorov-Smirnov test) and U Mann-Whitney test was used for comparison between genders.

Correlations among variables in serum, diet, MPOD and CT were evaluated using Spearman’s rho correlation coefficient. For the analysis of the correlations among variables and for the generalized linear model we used a total sample of n = 145 subjects. As to the n = 92 from this study we incorporated data from 53 subjects (26 women, 27 men), with the same characteristics, from a preliminary study previously published [20, 21].

Multiple linear regression analysis was carried out using backward elimination as a model selection procedure, with MPOD as dependent variable and as independent variables the lipids concentrations, the lutein and xanthophylls expressed in relation to lipids concentrations (cholesterol, HDL-cholesterol, LDL-cholesterol, serum lutein+zeaxanthin, lutein+zeaxanthin/HDL-, lutein+zeaxanthin/LDL-, lutein+zeaxanthin/cholesterol+TG) and, fruit intake and vegetable intake.

Correlations among CT and MPOD, fruit and vegetable intake and lutein+zeaxanthin/cholesterol+TG in serum were established using Spearman’s rho correlation coefficient. The statistical models used were generalized linear models (TWEEDIE distribution and LINK = LOG), with CT at 6 visual angle (size) stimuli, as the dependent variable and with fixed factor (gender) and covariates (serum lutein, HDL-cholesterol, LDL-cholesterol, cholesterol+ TG, MPOD and fruit +vegetables).

All reported P-values are based on a two-sided test and compared to a significance level of 5%. IBM SPSS v.25 Statistics software was used.

## Results

Table 1 shows subject characteristics, including lipid concentrations as possible confounding factor for the interpretation of the lutein and zeaxanthin status. There were no differences in age range and blood pressure. Regarding lipid profile, only the HDL-cholesterol showed differences between genders, being higher in women. No difference was found in the BMI but diferences were found in body fat percentage calculated from BMI and age data) which was higher in women than men. Most of the subjects did not smoke (81 out of 92).

**Table 1:** Characteristics and lipid profile (mmol/L).

	Total sample (n = 92)	Women (n = 68)	Men (n = 24)
Age (y)	54.5 ± 5.8 (55)	54.6 ±5.9 (55)	54.1 ± 5.4 (54)
BMI (kg/m^2^)	24.8 ± 3.1 (24.3)	24.6 ± 3.2 (24)	25.5 ± 2.9 (26)
Body fat (%)*	30.8 ±6.5 (31)	33.1 ±5.5 (32.6)	24.3± 4.3 (25.1)
Glucose (mmol/L)	4.48 ± 0.50 (4.44)	4.42 ± 0.44 (4.44)	4.65 ± 0.63 (4.83)
Cholesterol (mmol/L)	5.33 ± 0.75 (5.25)	5.38 ± 0.76 (5.26)	5.20 ± 0.71 (5.24)
HDL-cholesterol (mmol/L)	1.64 ± 0.37 (1.62)	1.72 ± 0.35 ^a^ (1.76)	1.41 ± 0.28 ^b^ (1.41)
LDL-cholesterol (mmol/L)	3.22 ± 0.66 (3.18)	3.19 ± 0.69 (3.13)	3.31 ± 0.57 (3.28)
Triglycerides (mmol/L)	1.00 ± 043 (0.88)	0.98 ± 0.44 (0.85)	1.06 ± 0.39 (1.01)
Cholesterol+TG (mmol/L)	6.33 ± 1.18 (6.13)	6.36 ± 1.2 (6.1)	6.26 ± 1.1 (6.25)
Diastolic pressure (mm Hg)	80 ± 1 (78,5)	79 ± 10.1 (77.5)	83.1 ± 9 (79.8)
Systolic pressure	125.8 ± 17.8 (122)	124 ± 17.8 (120)	131.4 ± 17.1 (125.5)

Mean ± standard deviation and (median).

^a,b^ Significant differences between genders (*p* = 0.000)

* Calculated indirectly from age and BMI data.

The lutein and zeaxanthin concentrations in serum and in dietary intake, MPOD, energy and fruit and vegetable consumption are shown in Table 2. Women exhibited significantly higher lutein concentrations in serum and higher MPOD levels but there were no differences in lutein and zeaxanthin intake or fruit and vegetable consumption. Serum lutein concentration related to LDL-cholesterol and to cholesterol plus TG were also higher in women than in men (*p* = 0.021 and *p* = 0.016, respectively). The selected serum lutein concentration cut-off point of 0.63 μmol/L for the volunteers included in this study, matched its 92 percentile.

**Table 2:** Serum concentrations and dietary intake of lutein and zeaxanthin and, MPOD [mean ± standard deviation and (median)].

	Total sample (n = 92)	Women (n = 68)	Men (n = 24)
***Serum concentrations***			
Lutein (μmol/L)	0.37 ± 0.12 (0.36)	0.39 ± 0.11 ^a^ (0.37)	0.33 ± 0.11 ^b^ (0.32)
Zeaxanthin (μmol/L)	0.10 ± 0.05 (0.08)	0.10 ± 0.04 (0.10)	0.11± 0.06 (0.12)
Lutein+zeaxanthin/cholesterol+TG (μmol/mmol)	0.09 ± 0.03 (0.09)	0.09 ± 0.03 (0.09)	0.08 ± 0.03 (0.08)
***Dietary intake***			
Lutein+zeaxanthin (μg/day)	1503 ± 1433 (1089)	1446 ± 1219 (1037)	1664 ± 1939 (1177)
Lutein+zeaxanthin (μg/1000 kcal/day)	724 ± 677 (520)	741 ±626 (548)	677 ± 819 (459)
Fruit intake (g/day)	375 ± 281 (323)	366 ± 261 (336)	400 ± 334 (240)
Vegetable intake	342 ± 150 (342)	342 ± 143 (342)	344 ± 173 (348)
Fruit+vegetable intake	717 ± 339 (650)	707 ± 313 (654)	744 ± 410 (649)
Energy intake (kcal/d)	2136 ± 299 (2073)	1990 ± 167 ^a^ (1960)	2550 ± 176 ^b^ (2503)
*Macular pigment optical density* (density units) (n = 184 eyes)	0.34 ± 0.13 (0.34)	0.35 ± 0.13 ^a^ (0.36)	0.31 ± 0.13 ^b^ (0.31)

^a,b^ Significant differences between genders: serum lutein (*p* = 0.019), energy intake (*p* = 0.000), MPOD (*p* = 0.034).

**Fig 1:**
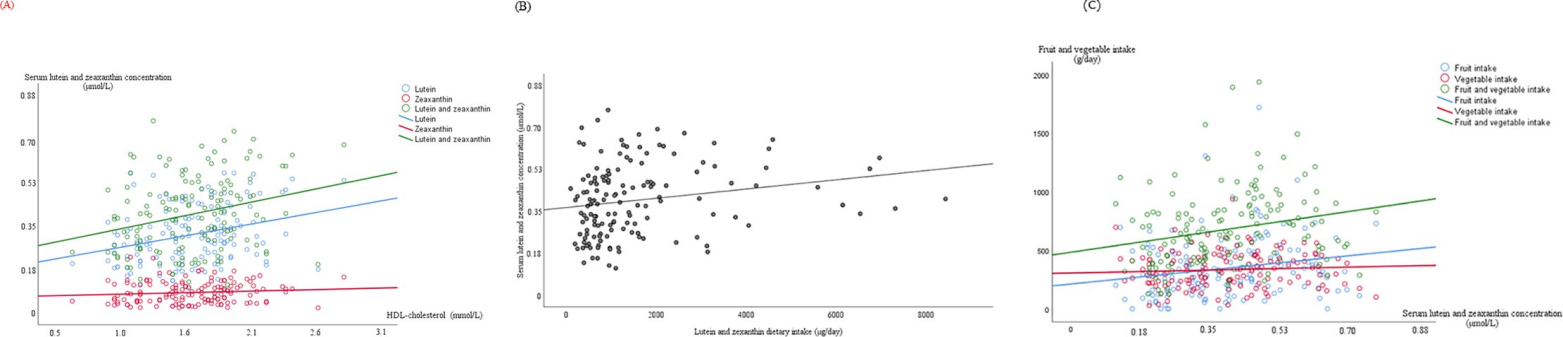
Statistically significant correlations between serum lutein and zeaxanthin concentrations with HDL-cholesterol. (A) and with their total dietary intake (B) and with their major food sources (C) (n = 145, 94 women and 51 men).

Significant correlations among serum lutein and zeaxanthin concentrations, HDL-chlesterol, their dietary intake and consumption of fruit and vegetables are shown in Fig 1. Serum lutein and zeaxanthin correlated with HDL-cholesterol, but not LDL-cholesterol. The same lipid variables correlated significantly with gender (HDL-cholesterol ρ = 0.421, *p*≤0,0001; cholesterol ρ = 0.122, *p* = 0.039). Gender also correlated with body fat (ρ = 0.656, *p*≤0,0001), which in turn correlates with HDL-cholesterol (ρ = 0.222, *p* ≤0.0001), with serum concentrations of lutein (rho = 0.216, *p*≤0.0001), zeaxanthin (rho = 0.130, *p* = 0.027), lutein plus zeaxanhin (rho = 0.187, *p* = 0.001), lutein+zeaxanthin/cholesterol+TG (rho = 0.206, *p*≤0.0001). The highest correlations were found between the lutein+zeaxanthin/1000 kcal intake and fruit and vegetable intake (rho = 0.525, *p*≤0.0001) and lutein+zeaxanthin intake with fruit and vegetables intake (rho = 0.482, *p*≤0.0001) (S1 Table).

**Fig 2:**
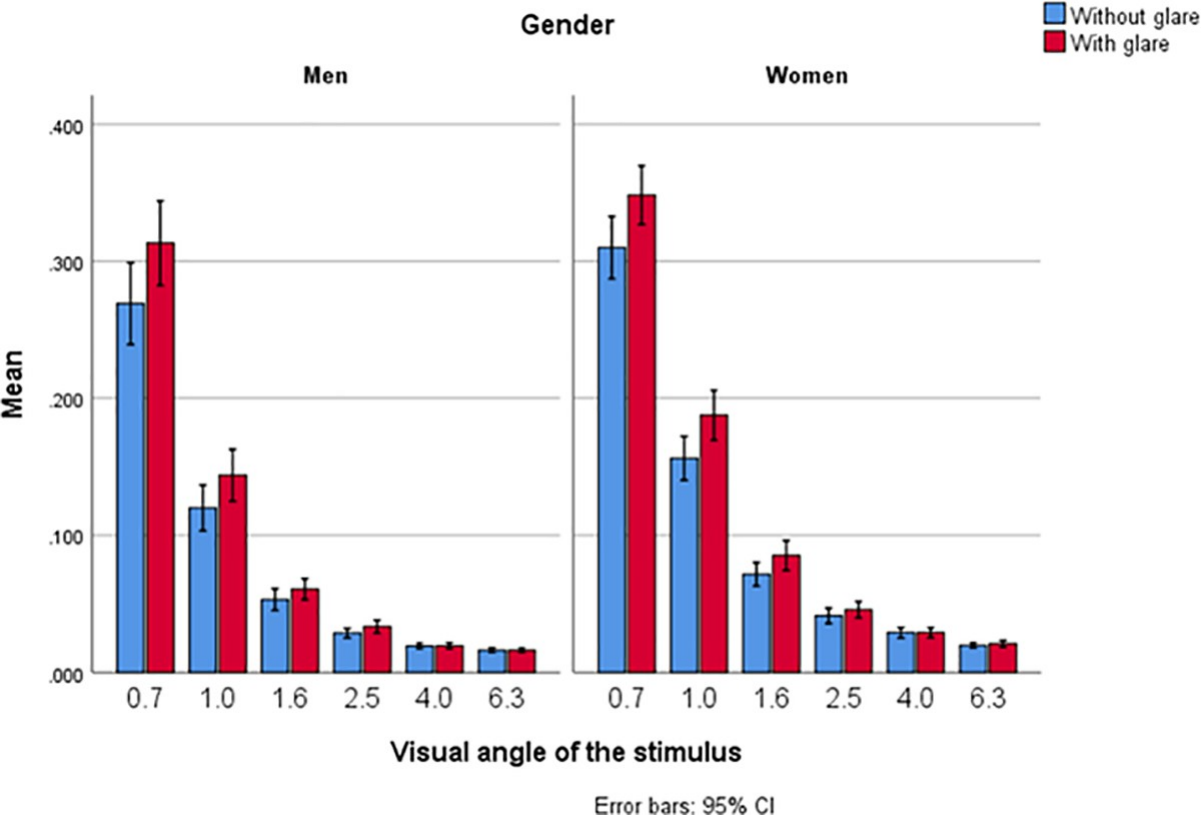
Contrast threshold at different degrees of visual angle, without and with glare (n = 290 eyes, 190 women and 100 men).

**Fig 3:**
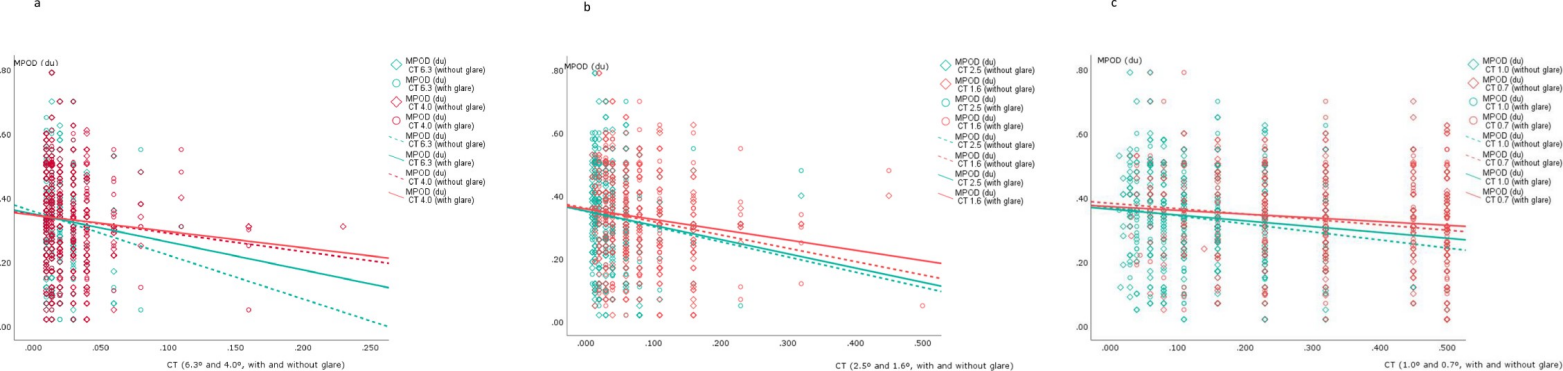
Statistically significant correlations (Spearmn’s rho, (*p* value) between MPOD (two eyes/subject, n = 290) and contrast threshold with and without glare (a, b, c).

**Fig 4:**
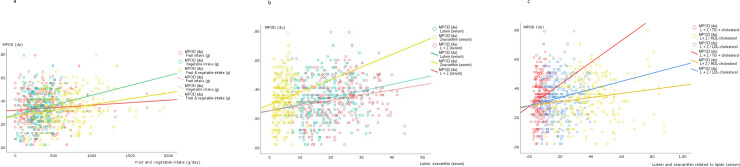
Statistically significant correlations (Spearmn’s rho, (*p* value) between MPOD (two eyes/subject, n = 290) and major food sources responsible for their intake. (a), serum lutein and zeaxanthin (b) and serum lutein and zeaxanthin related to lipids (c) (n = 145).

The CT data (means ± SD, medians and _95%_CI) for each of six different degrees of visual angle, with and without glare, are shown in Fig 2 (and S2 Table). Lower CTs imply better contrast sensitivity level at which a subject could detect each spatial frequency. The CT values were higher (worse) with glare than without glare at the medium and smaller visual angles (2.5°, 1.6°, 1.0°, 0.7°) but no differences were found for large stimulus sizes (6.3° and 4.0°). At most stimulus visual angles, CT negatively correlates with the MPOD (Fig 3). For its part, the MPOD correlates significantly with serum lutein and zeaxanthin concentrations and with fruit and vegetable consumption, but not with the lutein+zeaxanthin dietary intake (Fig 4). Moreover, HDL-cholesterol correlated only weakly with CT, and only with glare, at the large and medium stimulus sizes (at 6.3°: 0.160, p = 0.06; at 4.0°: 0.140, p = 0.017; at 2.5°: 0.116, p = 0.048). (S3 Table). None of the lipid profile variables correlated significantly with MPOD.

Table 3 shows the regression model used to evaluate the predictive value of serum lutein and zeaxanthin expressed in relation to serum lipids, fruit and vegetable consumption and gender, on the MPOD value. The table only shows results that do not include zero in the confidence interval. MPOD did not show diferences between genders in the total sample. Serum lutein plus zeaxanthin total concentration and serum lutein+zeaxanthin expressed in relation to HDL-cholesterol and, HDL-cholesterol, were the main predictors of MPOD. The R^2^ was 15.9% for the entire sample and, broken down by gender, was 17.6% and 23.2% for women and men, respectively. If the concentration of lutein+ zeaxanthin expressed in relation to lipids is not considered, the predictive value is only 8.4%, serum lutein being the main predictor.

**Table 3:** Multivariate regression analysis of biochemical and dietary factors and gender data (n = 145) associated with MPOD (n = 290).

	β (DE)	*P*≤	_95%_ CI
Constant	0.706 (0.109)	0.0001	0.492, 0.919
Lutein +zeaxanthin (serum)	0.023 (0.004)	0.0001	0.015, 0.031
Lutein+zeaxanthin/HDL	-0.907 (0.214)	0.0001	-1.328, -0.485
HDL-cholesterol	-0.00 (0.002)	0.0001	-0.009, -0.003

CI: confidence interval

Table 4 shows the results of the regression model (GELIN) used to assess the predictive value of gender, lutein serum concentration, lutein+zeaxanthin/cholesterol+TG, MPOD and fruit and vegetable consumption on the CT at six visual angles of different degress, with and without glare. MPOD and gender were predictors of CT at all visual angles and the main predictors of CT were the same with and without glare, although not at all visual angles as MPOD was only at medium and smaller angles (2.5°, 1.6°, 1.0° and 0.7°) and gender was not a predictor at 0.7°.

**Table 4:** Multivariate regression analysis of biochemical and dietary factors, gender (n = 145) and MPOD data associated with contrast threshold, without and with glare, (n = 290).

		Without glare			With glare		
Visual angle of the estimulus (^o^)		β (DE)	*p*≤	95% CI	β (DE)	*p*≤	95% CI
6.3	Intercept	-3.801 (0.074)	0.000	-3.946, -3.656	-3.740 (0.080)	0.000	-3.897, -3.583
	Gender	-0.186 (0.061)	0.002	-0.306, -0.066	-0.254 (0.067)	0.000	-0.386, -0.122
	MPOD	-0.392 (0.202)	0.052	-0.788, 0.003	-0.395 (0.218)	0.070	-0.823, 0.032
4.0	Intercept	-3.379 (0.094)	0.000	-3.563, -3.195	-3.415 (0.092)	0.000	-3.596, -3.235
	Gender	-0.393 (0.078)	0.000	-0.547, -0.240	-0.392 (0.078)	0.000	-0.545, -0.238
	MPOD	-0.513 (0.260)	0.048	-1.022, -0.005	-0.393 (0.252)	0.119	-0.888, 0.101
2.5	Intercept	-2.955 (0.101)	0.000	-3.154, -2.757	-2.866 (0.099)	0.000	-3.062, -2.671
	Gender	-0.359 (0.085)	0.000	-0.526, -0.193	-0.309 (0.084)	0.000	-0.475, -0.144
	MPOD	-0.723 (0.279)	0.010	-1.270, -0.175	-0.677 (0.273)	0.013	-1.212, -0.143
1.6	Intercept	-2.332 (0.101)	0.000	-2.531, -2.134	-2.177 (0.100)	0.000	-2.373, -1.981
	Gender	-0.292 (0.085)	0.001	-0.459, -0.126	-0.331 (0.085)	0.000	-0.499, -0.164
	MPOD	-0.950 (0.279)	0.001	-1.496, -0.404	-0.890 (0.276)	0.001	-1.430, -0.350
1.0	Intercept	-1.556 (0.098)	0.000	-1.748, -1.365	-1.444 (0.097)	0.000	-1.633, -1.255
	Gender	-0.257 (0.082)	0.002	-0.417, -0.096	-0.260 (0.081)	0.001	-0.419, -0.102
	MPOD	-0.938 (0.270)	0.001	-1.467, -0.410	-0.712 (0.264)	0.007	-1.230, -0.194
0.7	Intercept	-0.955 (0.085)	0.000	-1.121, -0.789	-0.926 (0.077)	0.000	-1.076, -0.776
	Gender	-0.134 (0.070)	0.055	-0.272, 0.003	-0.103 (0.062)	0.097	-0.225, 0.019
	MPOD	-0.672 (0.231)	0.004	-1.125, -0.219	-0.394 (0.208)	0.050	-0.801, 0,013

## Discussion

This expanded study focused on the complete sequence and correlations between markers of lutein and zeaxanthin dietary intake and status (serum concentrations and MPOD), lipid profile and the CT as the potential functional effect of lutein and zeaxanthin, in subjects with well defined characteristics and eating their normal diet. Comparing these findings with previous results from subjects with the same characteristics (age, BMI, lipid concentrations) [20], observed that serum lutein and zeaxanthin concentrations were higher (median concentrations of lutein: 0.361 *vs* 0.0242 μmol/L, zeaxanthin: 0.078 *vs* 0.048 μmol/L). This could be attributable to the higher dietary intake of lutein+zeaxanthin (median value 1089 *vs* 679 μg/d) and of fruits and vegetables intakes (major contributors to their dietary intake; median fruit intake 323 *vs* 269 g/d, vegetable 342 vs 291 g/d) in this study. MPOD was slightly higher (0.34 *vs* 0.32 du) but no gender difference was found in the overall sample. Although we found only a minor correlation between these lutein and zeaxanthin markers and visual function in a previous study of healthy adults [20], in a different study of subjects with similar characteristics (age, BMI) but higher MPOD values, a significant correlation between MPOD and the disability glare threshold was reported [12].

A comparison of our data of lutein plus zeaxanthin dietary intake (1.5 mg/d) and serum concentration (0.447 μmol/L) data withthe ranges found in studies included in a recen review, showed that both markers fall in the mean range reported by Böhm [16] for dietary intake (0.06–4.84 mg/d) and serum concentration (0.199–0.561 μmol/L). The lutein+zeaxanthin dietary intake observed in this study is much lower than that associated with desecreased risk of several chronic diseases (6 mg lutein/d) [5, 19, 28]. Moreover, serum concentration was also lower than that associated with lower risk level in epidemiological studies and with improvement in physiological functions and thus, quality of life (> 0.63 μmol/L) [5].

We obtained a similar or high correlations between lutein and zeaxanthin concentrations in the dietary intake and serum (r = 0.333) to that found in a systematic review of more than a hundred studies (r = 0.29, _95%_CI: 0.26, 0.32) [29]. Interestingly, serum lutein and zeaxanthin concentration correlated significantly with fruit consumption (r = 0.240) but not with the vegetable consumption. This occurred despite the fact that the amount of lutein+zeaxanthin supplied by vegetables is much greater than that supplied by fruits [20, 30]. This higher correlation between serum lutein and zeaxanthin concentrations with fruit intake could be partially by the fact that these xanthopylls are found in fruits in ester forms where bioavailability is equivalent or even higher than that of free forms [31]. However, dietary intake of lutein and zeaxanthin (total intake and lutein+zeaxanthin/1000 Kcal) correlated more closely with vegetable as opposed to fruit consumption.

We found that MPOD correlated significantly with the fruit and vegetable consumption and with lutein and zeaxanthin serum concentration, as also described in other studies of subjects of the same age range [20, 32, 33] and with CT as the final visual outcome marker. Many other factors come into play in these correlations such as gender, age, smoking status, circulating lipids, carotenoid intake patterns and BMI [15], to name just a few, and these should be taken into account to draw accurate comparisons between studies. More relevant than BMI is body fat percentage since a high proportion of the carotenoids in the body are found in adipose tissure and an inverse correlation was found between macular pigment density and body fat in obese subjects [34] and in subjects with relatively healthy body fat limits [35]. In our study, a similar percentage of body fat in non-obese subjects (24.6% and 31.9%, for men and women respectively, whole sample median 31%), exhibited an inverse, but not statistically significant correlation with MPOD. Surprisingly, body fat correlated with HDL-cholesterol (rho = 0.222, *p*≤0.0001), but not with LDL-cholesterol, and both body fat and HDL-cholesterol were higher in women than in men.

The lipoprotein profile is likely to affect MP levels and, although the mechanisms controlling the deposition and stabilization of MP are not fully understood [16], they are believed to be regulated by binding proteins such as the scavenger receptor class B type-1 (SR-B1) that acts as an HDL cell surface receptor [15, 36]. Moreover, dietary n-3 fatty acids (mainly DHA) seem to have a significant impact on macular lutein and zeaxanthin uptake [17, 37]. Although, lutein and zeaxanthin are transported by lipoproteins in the blood and could be evenly distributed between LDL and HDL fractions [15, 38], a selective uptake from HDL-cholesterol by retinal pigment epithlium has been described [39]. In this study, serum lutein and zeaxanthin correlated with cholesterol and HDL-cholesterol, but not with LDL-cholesterol, which is consistent with the majority presence of these carotenoids in HDL-cholesterol compared to LDL-cholesterol described in several studies [32, 36, 40–43]. However, HDL and LDL transport these carotenoids in very different proportions: LDL carries about one molecule per LDL particle while HDL carries only one carotenid molecule in every twenty HDL particles [36].

MPOD has been associated with HDL-cholesterol in young healthy subjects (mean age: 23) [1] but no associations was reported for wider age range (21–66 and 18–75) [41, 43, 44]. While, we did not find any correlation between MPOD and serum cholesterol or lipoproteins, we did find a correlation with serum lutein+zeaxanthin concentration expressed in relation to lipoproteins. Differences between studies that could account for inconsistencies in results include sample size, MP measurements methods and two other important aspects that are generally overlooked: age group and cholesterolemia [20].

The main predictor of the MPOD in this study was the lutein+zeaxanthin/HDL-cholesterol concentration, which exhibited an inverse association. Thus high MPOD was associated with a lower lutein+zeaxanthin/HDL-cholesterol ratio than that for the same serum lutein+zeaxanthin concentration obtained with a higher HDL-cholesterol concentration. Hence, body fat percentage should be taken into account as adiposity may affect the nutritional status of the retina [35]. A correlation between HDL-cholesterol, gender and body fat was found therefore warranting a more precise study estimating body fat directly.

A high HDL-cholesterol concentration is associated with cardiovascular health [36], but is also associated with a higher risk of ARMD [45, 46]. The conflicting correlations between the circulating lipoprotein concentrations and ARMD could be partly due to changes that HDL particles undergo in terms of their composition and biological properties under different physiological and pathological conditions [47].

The coefficient of determination of MPOD for serum lutein and zeaxanthin concentrations when expressed in relation to HDL-cholesterol was 15.9% for the entire sample (23.2% in men, 17.6% in women), which is lower than the 29.7% obtained in the previous study [20]. Although in both studies serum lutein and zeaxanthin concentrations and lutein+zeaxanthin expressed in relation to lipids were the determining factors of the MPOD, in the previous study the lipids were cholesterol+TG but in the present study was HDL-cholesterol. Although we cannot explain these different results, this study included about three times more women than men while the same number of men and women participated in the previous study. In both studies, higher HDL-cholesterol and higher body fat were found in women than in men. Of the different posible explanations, greater variability in lutein and zeaxanthin uptake by subjects’ ocular tissue can not be ruled out. In anycase, for a better interpretation of the results, lutein and zeaxanthin concentrations should be expressed in relation to lipids.

It is important to not only know how to modify MPOD through diet, but also the degree to which MPOD correlates to vision in apparently healthy subjects. MPOD can optimize visual performance because of its pre-receptoral absorption of blue light and the subsequent attenuation of the effects of chromatic aberration and the adverse effect of light scatter [36, 48–50]. Increased MPOD has been associated with improved visual function in patients with early stage AMD [51, 52] and in control subjects [12, 53], In turn, improved visual function has been associated with higher serum lutein and zeaxanthin concentrations in patients with cataracts [54].

Although, most studies evaluating lutein and zeaxanthin intake and visual outcomes focus on MPOD, there are some that use other visual outcome indicators (contrast sensitivity, glare sensitivity, photostress recovery, visual acuity) which need to be thoroughly studied in healthy individuals with a varied diet [4]. In a group of these apparently healthy individuals consuming varied diets who participated in the present study, their MPOD showed stronger association with CT than in the previous study of subjects in the same age range [20], mainly at medium and smaller visual angle degrees which correspond to mediun and high spatial frequencies. Sensitivity declines with age for medium and high frequencies [55], leading to a reduction in visual acuity.

The main strengths of this study were the nutritional approach used to detect the correlation between lutein and zeaxanthin habitual dietaty intake (excluding lutein/ zeaxanthin supplements) on the final visual outcome (contrast threshold) using a wide range of markers (in dietary intake, serum concentration and MPOD), the well-characterized healthy individuals meeting relevant criteria and the use of a specific carotenoid food composition table containing data generated by our group and considered as highly acceptable [56]. The main limitations were mainly the absence of gold standard techniques or test parameters to assess visual performance. Although the technique and reliability of the MPOD device were described in detail by van der Veen [27], it was difficult to compare results across studies. Moreover, the self-reported ocular health of the participants (not accurately controlled) and there was a gender imbalance (nearly double number of women than men).

## Conclusions

Serum lutein and zeaxanthin and fruit and vegetable consumption (and not lutein and zeaxanthin from habitual dietary intake) in our sample population of middle-aged Spanish subjects was associated with MPOD, which in turn, was a predictor of contrast sensitivity. However, significant associations were also found in healthy normolipemic subjects with respect to HDL cholesterol (and not LDL cholesterol), further associated with serum lutein and zeaxanthin concentrations and MPOD, which, in turn, determines visual potential (lower CT and thus higher contrast sensitivity). These findings are increasingly important to potentially optimize the visual status of an ageing society. Because of the crucial role that xanthophyll carotenoids and essential fatty acids are known to play in health and disease, our data has implications beyond healthy individuals, age 45–65, who are the target population for supplements marketed to improve vision quality and reduce the risk of chronic eye disease and who could benefit from recommendations regarding carotenoid intake or desired blood or tissue concentrations to improve ocular health. Future studies should move well beyond our cross-sectinal data and focus on the progression of disease (i.e. macular degeneration, cataracts and cognitive dysfunction) associated with lipid effects on carotenoids.S1.Data

## Supporting information

S1 Table Statistically significant correlations (Spearman’s rho, (*p* value) between lutein, zeaxantin, lipids and major food sources for dietary intake and serum concentrations (n = 145, 94 women and 51 men). (DOCX)

S2 Table Contrast threshold at different degrees of visual angle, without and with glare (n = 290 eyes, 190 women and 100 men). (DOCX)

S3 Table Statistically significant correlations (Spearmn’s rho, (pvalue) between MPOD (two eyes/subject, n = 290).and lutein, zeaxanthin and major food sources for their intake in serum and diet (n = 145) and contrast threshold (n = 290). (DOCX)

S1 Data (SAV)
