# Supplementary material for: Predictors of macular pigment and contrast threshold in Spanish healthy normolipemic subjects (45–65 years) with habitual food intake
Source: PLoS One. 2021 May 27;16(5):e0251324. doi: 10.1371/journal.pone.0251324 (PMC8159008; doi:10.1371/journal.pone.0251324)
Supplement: S1 Table — (DOCX) [file pone.0251324.s001.docx]

S1 Table. Statistically significant correlations (Spearman’s rho, (*p* value) between lutein, zeaxantin, lipids and major food sources for dietary intake and serum concentrations (n=145, 94 women and 51 men)

|  | Serum | | | | | | | | Diet | | | | |
| --- | --- | --- | --- | --- | --- | --- | --- | --- | --- | --- | --- | --- | --- |
|  | Lutein | Zeax. | | Lutein+Zeax. | Lutein+Zeax. /chol.+TG | Cholesterol+TG | | Lutein+Zeax. | | | Lutein+zeax./  1000 kcal | |  |
| *Serum concentrations* | | | | | | | | | | | | | |
| HDL-cholesterol | 0.274  (0.001) | |  | 0.253  (0.002) |  |  |  | | |  | |  |  |
| *Dietary intake* | | | | | | | | | | | | | |
| Lutein+Zeax. | 0.363  (<0.0001) | |  | 0.333  (0.001) | 0.358  (<0.0001) |  |  | | |  | |  |  |
| Lutein+Zeax./ 1000 kcal | 0.376  (<0.0001) | |  | 0.331  (<0.0001) | 0.366  (<0.0001) |  |  | | |  | |  |  |
| Fruit | 0.233  (0.005) | |  | 0.240  (0.004) | 0.283  (0.001) | -0.208  (0.012) | 0.353  (0.001) | | | 0.395  (<0.0001) | |  |  |
| Vegetable |  | | 0.164  (0.048) |  |  |  | 0.481  (<0.0001) | | | 0.504  (<0.0001) | |  |  |
| Fruit+vegetable | 0.281  (0.001) | | 0.195  (0.019) | 0.281  (0.001) | 0.306  (<0.0001) |  | 0.482  (<0.0001) | | | 0.525  (<0.0001) | |  |  |
