# Supplementary material for: Predictors of macular pigment and contrast threshold in Spanish healthy normolipemic subjects (45–65 years) with habitual food intake
Source: PLoS One. 2021 May 27;16(5):e0251324. doi: 10.1371/journal.pone.0251324 (PMC8159008; doi:10.1371/journal.pone.0251324)
Supplement: S2 Table — (DOCX) [file pone.0251324.s002.docx]

S2 Table. Contrast threshold at different degrees of visual angle, without and with glare (n=290 eyes, 190 women and 100 men).

|  | Total sample | | Women | | Men | |
| --- | --- | --- | --- | --- | --- | --- |
| Visual angle of the stimulus (^o^) | Without glare | With glare | Without glare | With glare | Without glare | With glare |
| 6.3 | 0.018 ± 0.011  (0.014)  [0.017 , 0.020] | 0.019 ± 0.014  (0.014)  [0.018 , 0.022] | 0.020 ± 0.012  (0.014)  [0.020 , 0.021] | 0.021 ± 0.016  (0.014)  [0.018 , 0.023 ] | 0.016 ± 0.008  (0.014)  [0.015 , 0.018] | 0.016 ± 0.007  (0.014)  [0.015 , 0.018] |
| 4.0 | 0.026 ± 0.022  (0.020)  [0.023 , 0.028] | 0.024 ± 0.020  (0.020)  [0.023 , 0.028] | 0.029 ± 0.026  (0.020)  [0.025 , 0.033] | 0.029 ± 0.025  (0.020)  [0.025 , 0.033] | 0.019 ± 0.010  (0.014)  [0.017 , 0.021] | 0.019 ± 0.011  (0.014)  [0.017 , 0.022] |
| 2.5 | 0.037 ± 0.034  (0.030)  [0.032 , 0.042] | 0.041 ± 0.036  (0.030)  [0.037 , 0.046] | 0.041 ± 0.039  (0.030)  [0.036 , 0.047] | 0.046 ± 0.041  (0.030)  [0.040 , 0.052] | 0.029 ± 0.017  (0.030)  [0.050 0.032] | 0.033 ± 0.023  (0.030)  [0.029 , 0.038] |
| 1.6 | 0.065 ± 0.055  (0.040)  [0.059 , 0.072] | 0.077 ± 0.067  (0.060)  [0.069 , 0.084] | 0.072 ±0.060  (0.060)  [0.063 , 0.080] | 0.085 ± 0.076  (0.060)  [0.074 , 0.096] | 0.053 ± 0.040  (0.040)  [0.045 , 0.045] | 0.061 ± 0.039  (0.060)  [0.053 , 0.068] |
| 1.0 | 0.144 ± 0.105  (0.110)  [0.132 , 0.156] | 0.172 ± 0.119  (0.160)  [0.159 , 0.186] | 0.156 ± 0.112  (0.110)  [0.140 ,0.172] | 0.188 ± 0.127  (0.160)  [0.169 , 0.206] | 0.120 ± 0.084  (0.110)  [0.103 , 0.137] | 0.144 ± 0.095  (0.110)  [0.125 , 0.163] |
| 0.7 | 0.296 ± 0.157  (0.320)  [0.278 , 0.314] | 0.336 ± 0.152  (0.320)  [0.319 , 0.354] | 0.310 ± 0.159  (0.320)  [0.287 ,0.333] | 0.348 ± 0.150  (0.320)  [0.327 , 0.370] | 0.269 ± 0.150  (0.230)  [0.239 , 0.299] | 0.313 ± 0.155  (0.320)  [0.282 , 0.344] |

Values are expressed as means ± SD, (medians) and [_95%_CI]
