# Supplementary material for: Predictors of macular pigment and contrast threshold in Spanish healthy normolipemic subjects (45–65 years) with habitual food intake
Source: PLoS One. 2021 May 27;16(5):e0251324. doi: 10.1371/journal.pone.0251324 (PMC8159008; doi:10.1371/journal.pone.0251324)
Supplement: S3 Table — (DOCX) [file pone.0251324.s003.docx]

S3 Table. Statistically significant correlations (Spearmn’s rho, (pvalue) between MPOD (two eyes/subject, n=290).and lutein, zeaxanthin and major food sources for their intake in serum and diet (n=145) and contrast threshold (n=290).

|  | MPOD |
| --- | --- |
| Lutein (serum) | 0.226 (<0.0001) |
| Zeaxanthin (serum) | 0.177 (0.003) |
| Lutein+zeaxanthin (serum) | 0.229 (<0.0001) |
| Lutein+zeax./chol.+TG | 0.165 (0.005) |
| Lutein+zeax./LDL | 0.159 (0.007) |
| Lutein+zeax./HDL | 0.149 (0.011) |
| Vegetable intake | 0.163 (0.005) |
| Fruit +vegetable intake | 0.202 (0.001) |
| *Contrast threshold –visual angle of the stimulus* | |
| *Without glare* |  |
| 6.3 | - 0.121 (0.039) |
| 4.0 | -0.160 (0.006) |
| 2.5 | -0.182 (0.002) |
| 1.6 | -0.205 (<0.0001) |
| 1.0 | -0.193 (0.001) |
| 0.7 | -0.201 (0.001) |
| *With glare* |  |
| 6.3 | -0.135 (0.022) |
| 4.0 |  |
| 2.5 | -0.155 (0.008) |
| 1.6 | -0.152 (0.009) |
| 1.0 | -0.160 (0.006) |
| 0.7 | -0.142 (0.015) |
